# Formative evaluation of a telemedicine model for delivering clinical neurophysiology services part I: Utility, technical performance and service provider perspective

**DOI:** 10.1186/1472-6947-10-48

**Published:** 2010-09-15

**Authors:** Patricia Breen, Kevin Murphy, Geraldine Browne, Fiona Molloy, Valerie Reid, Colin Doherty, Norman Delanty, Sean Connolly, Mary Fitzsimons

**Affiliations:** 1 Epilepsy Programme, Beaumont Hospital, Dublin 9, Ireland; 2 Department of Neurology, Sligo General Hospital, Sligo, Ireland; 3 Department of Clinical Neurophysiology, Beaumont Hospital, Dublin 9, Ireland; 4 Department of Neurology, St. James's Hospital, James's Street, Dublin 8, Ireland; 5 Department of Clinical Neurophysiology, St. Vincent's University Hospital, Elm Park, Dublin 4, Ireland

## Abstract

**Background:**

Formative evaluation is conducted in the early stages of system implementation to assess how it works in practice and to identify opportunities for improving technical and process performance. A formative evaluation of a teleneurophysiology service was conducted to examine its technical and sociological dimensions.

**Methods:**

A teleneurophysiology service providing routine EEG investigation was established. Service use, technical performance and satisfaction of clinical neurophysiology personnel were assessed qualitatively and quantitatively. These were contrasted with a previously reported analysis of the need for teleneurophysiology, and examination of expectation and satisfaction with clinical neurophysiology services in Ireland. A preliminary cost-benefit analysis was also conducted.

**Results:**

Over the course of 40 clinical sessions during 20 weeks, 142 EEG investigations were recorded and stored on a file server at a satellite centre which was 130 miles away from the host clinical neurophysiology department. Using a virtual private network, the EEGs were accessed by a consultant neurophysiologist at the host centre for interpretation. The model resulted in a 5-fold increase in access to EEG services as well as reducing average waiting times for investigation by a half. Technically the model worked well, although a temporary loss of virtual private network connectivity highlighted the need for clarity in terms of responsibility for troubleshooting and repair of equipment problems. Referral quality, communication between host and satellite centres, quality of EEG recordings, and ease of EEG review and reporting indicated that appropriate organisational processes were adopted by the service. Compared to traditional CN service delivery, the teleneurophysiology model resulted in a comparable unit cost per EEG.

**Conclusion:**

Observations suggest that when traditional organisational boundaries are crossed challenges associated with the social dimension of service delivery may be amplified. Teleneurophysiology requires a governance and management that recognises its socio-technical nature.

## Background

In Ireland, as in many countries, the health service is undergoing a programme of reform aimed at improving patient care, providing better value for money and enhancing healthcare management [1]. Implementation of telemedicine technologies has the potential to support objectives of the reform programme which include provision of consistent national, regional and local patient-centred care and development of a health system which maximises the use of resources by delivering the right care in the right setting. An example of this is the use of telemedicine to deliver clinical neurophysiology (CN) services.

CN is an essential subspecialty of clinical neuroscience and provides the most widely used tests to evaluate the integrity and function of the nervous system. CN investigation includes electroencephalography (EEG), electromyography (EMG), nerve conduction studies (NCS) and evoked potential (EP) recording. Using specialised electromedical equipment, small amplitude bioelectric signals originating in the brain, along nerve pathways or in muscles are recorded via electrodes which are placed in contact with the patient undergoing the investigation. The traditional model of service delivery requires the patient to attend the CN department in person. Contemporary CN technology stores recorded signals in digital form and is compatible with teleneurophysiology service development.

A comprehensive analysis of CN in Ireland has previously been reported [2,3] and included a retrospective audit of referrals to six CN departments as well as surveys of satisfaction with CN services. A total of 4954 referrals for EEG, EMG, NCS and EP over a 12 month period were examined and demonstrated an unmet demand for CN services in Ireland with residents beyond large urban centres being further disadvantaged. Identified geographical inequities included access to CN, referral pathways, waiting list delays, and age profile of CN patients. Furthermore, referring clinicians believed that distance and long waiting lists either deterred or made referral irrelevant. That exploratory study proposed a telemedicine model of service delivery to enhance responsiveness and improve access to care. To determine the effectiveness of the proposed solution a teleneurophysiology service which provided routine EEG investigations was established. A formative evaluation of the model is presented here and in an accompanying paper [4].

**Figure 1:**
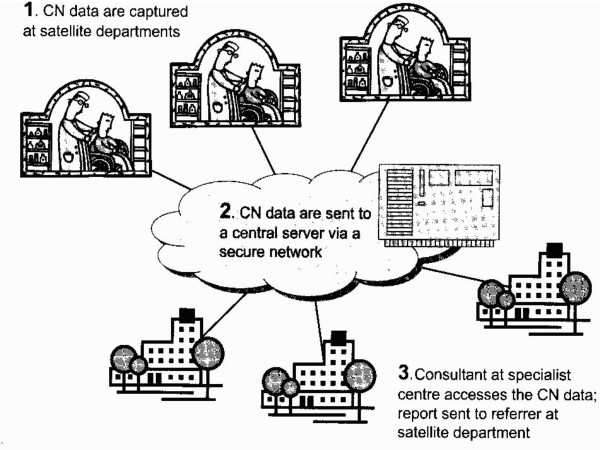
**The telemedicine model of clinical neurophysiology service delivery**. CN = Clinical neurophysiology (*from Connolly S, Fitzsimons M. 2005*).

The first example of tele-EEG was reported more than 30 years ago [5]. At that time analogue EEG signals were transmitted over standard telephone lines. The advent of digital CN equipment interfaced with high-speed telecommunications infrastructure has further enhanced the clinical service possiblities [6-11]. In the teleneurophysiology model a technologist records bioelectric data from patients at a satellite CN clinic. Using a secure network, data is transferred to a central server for storage from where it can be accessed for interpretation by an authorised consultant neurophysiologist at a specialist CN centre (See Figure 1) [8]. The result of the investigation can then be conveyed to the referring clinician. In keeping with the European Commission's definition of telemedicine this model of service delivery can result in rapid access to shared and remote CN expertise by means of telecommunications and information technologies, no matter where the patient, clinician or relevant information is geographically located.

While telemedicine models can benefit the structure and process of health service delivery, less is known about its long term economic benefit and effect on patients' health [12-20]. Bashshur et al., (2005) propose a number of reasons for this including the fact that funding for telemedicine generally goes towards programme development without complementary funding for related evaluation research. In addition, the type of measurement data available for analysis, and hence the type of evaluation, will vary with the developmental stage or point in the life-cycle of the telemedicine service. Formative or constructive evaluation is appropriate in the early stages of an implementation which aims to examine operational aspects and identify any changes to the system that are required to achieve stable performance. When the structures and processes are optimized, summative evaluation becomes relevant and focuses on the measurement of outcomes such as changes in health status, quality of life, and cost-benefit analysis [12,13,17,21]. Summative evaluation requires longer time-frame to produce these effects.

Previously reported studies, conducted during the technical development or early adoption phase of tele-EEG, have demonstrated its technical feasibility together with healthcare provider and patient satisfaction with the system [5-7,9-11,21]. Brender [21] suggests that the next step in evaluating health informatics sytems should take place during the adaptation phase. This is the period immediately after a system has been put into operation and during which technical and process adjustments may be required to achieve stability. Therefore, the study presented in this paper is a formative assessment conducted in the adaptation phase [21] of a teleneurophysiology service to test the effectiveness of a telemedicine model of CN service delivery within bounded conditions.

Based on routine EEG service, the objectives of this study were to evaluate the teleneurophysiology model in terms of utility, technical performance, service provider (CN personnel) satisfaction and potential for cost benefit. Any observed operational difficulties might suggest a need to adjust operating procedures. It was intended that this study would further our understanding of resources, protocols and procedures required to deliver and govern such services and could contribute to future policy regarding the development of teleneurophysiology services in Ireland. In an accompanying paper we report on referring clinician and patient satisfaction with the model [4].

## Methods

A two-step quasi-experimental design was used to evaluate the adaptive phase of a teleneurophysiology intervention [21-24]. Using a one-group posttest-only approach, post-intervention observations were made without reference to any control group or control period [22]. Additionally, a one-group pretest-posttest strategy compared post-intervention observations with data from a control period. These control period data were accessed from a previously reported analysis of need for teleneurophysiology in Ireland [2,3]. Both qualitative and quantitative data was collected and analysed. The medical research ethics committees of both Beaumont Hospital, Dublin and Sligo General Hospital reviewed and approved the study.

### Context of the study

#### a) Routine EEG investigation

This study concerned the delivery of a routine EEG service. Routine EEG testing is commonly used in the investigation of epilepsy and other seizure disorders and involves a 20 - 40 minute recording of around 20 channels of time varying electrical activity of the brain via a set of electrodes attached to the scalp. Modern EEG recordings are accompanied by a synchronously recorded digital video. This is useful if the patient has a clinical event during the test as clinical features captured on video can be correlated with the EEG for interpreting and reporting purposes. The digital file sizes generated are approximately 35 MB (megabytes) for the EEG component and 350 MB for the associated video. Generally the video component of the file is edited by the recording technologist so that only the clinically relevant segments of the video are retained.

#### b) Organisational setting

Beaumont Hospital located in Dublin, the capital of Ireland, is a tertiary referral centre for clinical neurosciences in Ireland. Sligo General Hospital (SGH) and Letterkenny General hospital (LGH) are two secondary referral facilities serving a population of more than 220,000 in the northwest region of Ireland. At the time of this study there were no specialist neurology or CN services in the region. To test the telemedicine model, the central hub of a teleneurophysiology service was established at the clinical neurophysiology department of Beaumont Hospital while a satellite centre was set up at SGH. The distance from Dublin to SGH is 130 miles.

### Study participants

#### a) Service providers

This study investigated the telemedicine model from the CN service provider perspective. In this regard, participants included 2 neurophysiological measurement technologists who recorded the EEGs at SGH and 1 consultant in CN who interpreted the EEGs at Beaumont Hospital.

#### b) Patients

##### Inclusion criteria

• Patients being investigated for neurological conditions such as suspected seizure disorders, cognitive changes, syncope are eligible for routine EEG recording.

• Patients will be over 18 years of age, regardless of race and gender.

• Written informed consent signed and dated by the patient or their legally accepted representative.

• No contraindications to having an EEG investigation

##### Exclusion criteria

• Contraindications to undergoing an EEG

• Patients < 18years of age

• Patients who will have difficulty co-operating with the EEG recording procedure.

### System details

A NicoletOne Clinical EEG system from Viasys Healthcare was installed at SGH to record EEGs with synchronous video. For reviewing the EEGs, a NicoletOne Review station was installed at Beaumont Hospital. Both systems consisted of a desktop computer with Windows XP Professional operating system. The recording equipment was cart mounted and included EEG and video acquisition software, 32 channel EEG amplifier, and deskjet printer. The review station included review software.

A virtual private network (VPN) established to provide a private, secure network for data transfer between Ireland's government agencies was exploited for this project. A VPN affords several layers of security including local agency firewalls, local passwords, site security policies, dedicated internet protocol (IP) addresses and encryption. A 45 Mb (megabit) link to the government VPN (GVPN) was available at SGH and a 100 Mb connection to the network existed at Beaumont. The EEG recording equipment was interfaced to a CN file server at SGH which in turn was interfaced to the GVPN. At Beaumont Hospital the review station was also routed to the GVPN. The configuration of the system allowed only authorised personnel at both hospitals to access the CN file server at SGH.

### The teleneurophysiology process

The suggested capacity for EEG services is 3.2 per 1000 population per year [2]. For the population of the northwest of Ireland approximately 700 EEG referrals per year would be expected and given the age profile of the Irish population [25] 525 (75%) of these would be for individuals aged 18years and over. We therefore anticipated a 10 per week maximum referral rate to the teleneurophysiology pilot. It was considered that a period of twenty weeks would allow for reasonable diffusion of the service, adequate time for potential referring clinicians to utilise it and sufficient time to make observations on its utility, technical performance and service provider satisfaction.

Over the twenty week teleneurophysiology pilot project, referrals for routine adult EEGs were accepted at SGH. Healthcare managers and clinicians in the northwest region were given six months notice in writing that the service would become available. Two months before the go-live date further information sessions were held at both LGH and SGH for potential referring clinicians. During the pilot phase, neurophysiological measurement technologists were present at SGH to record EEGs two days per week. The service was available to referring clinicians in the northwest region of Ireland who required EEG investigation for their patients.

The process of referral to the teleneurophysiology service was similar to the procedure followed in the traditional model. Requests for an EEG were made on a paper-based teleneurophysiology referral form. An administrative assistant at SGH scanned the referral forms into a "referrals" folder on the CN file server at SGH.

Via the GVPN a consultant neurophysiologist at Beaumont Hospital evaluated the referrals. A scheduling assistant also based at Beaumont Hospital arranged appointments for EEG recording at SGH. Appointment details together with directions to the EEG centre at SGH were sent by post to the patient. An information sheet describing the EEG procedure and the teleneurophysiology project were also included in the letter to the patient. The appointment details were also uploaded to an electronic schedule which was accessible to the teleneurophysiology personnel at SGH.

On arrival at SGH the patient had the opportunity to further discuss the investigation with the CN technologist and if happy to proceed the EEG recording was carried out. The recorded EEG with its synchronised digital video data was routed to the CN file server at SGH. From there it was available for interpretation by the Beaumont Hospital based consultant via the GVPN.

To review the EEG data stored on the SGH file server, the consultant at Beaumont Hospital directly accessed the relevant file via the GVPN i.e. without transferring it between sites. This ensures that annotations added to the record by the reviewer are saved with the primary EEG file and avoids the risk of incomplete records. It also eliminates unnecessary duplication of sensitive patient data. Generated reports were conveyed in hardcopy by post back to the referring clinician and a digital copy was also stored on the CN file server.

### Evaluation metrics

#### i) Utilisation data

Data on the utilisation of the teleneurophysiology service were audited including number of patients referred, patient age, gender, distance travelled by patient to SGH, referring clinician specialty, waiting time for appointment, reason for referral, and result of EEG test.

#### ii) Technical performance and service provider satisfaction

Using a mixture of feedback group meetings, frequent one-to-one conversations, and questionnaires which were to be completed after each recording or review session, data about satisfaction with and technical performance of the teleneurophysiology model was collected from the CN technologists and the consultant (table 1). Through these methods the performance of the EEG recording and review equipment, the CN local area network at SGH in terms of EEG file transfer to the file server, and the accessibility of EEG files at Beaumont Hospital via the GVPN was recorded. They were also asked to describe the nature of any problems encountered in the system. Satisfaction of the CN personnel with: the adequacy of referral information provided by referring clinicians, the EEG recording environment, quality of EEG recording, time taken to review EEGs, the EEG scheduling process, and communication between the host and satellite centres was documented.

**Table 1:** Topics discussed with CN personnel at feedback meetings and considered in satisfaction questionnaire.

For technologists:
*Referral details*
1. Were all sections of the EEG referral form complete?

*EEG recording*
2. Did you have any problems with the EEG recording equipment?
2a. Describe the nature of any problem encountered.

*EEG file transfer*
3. Did you transfer EEG files to file server storage?
3a. Did you experience any difficulties with file transfer?
3b. Describe the nature of any difficulty experienced.

*General comments*
4. Please provide any other comments about the teleneurophysiology recording session.

**For the consultant.:**

*Referral details*
1. Was all referral information available for the EEG reporting session
1a. Were all sections of the referral form complete?
1b. On a scale of 1 - 5 how appropriate do you think the referral is? (1 = referral was not appropriate for EEG investigation; 5 = referral was highly appropriate for EEG investigation

*EEG file access*
2. Was the EEG file accessible from the Beaumont EEG review station?
2a. Did you experience any difficulty in accessing required EEG files?
2b. Describe the nature of any difficulty encountered.

*EEG reporting*
3. How long did EEG reporting take - from accessing the EEG file to closing the EEG file?
3a. Did you encounter any problems with the EEG review equipment?
3b. Describe the nature of any difficulty encountered.
3c. On a scale of 1 - 5 please rate the quality of EEG recordings (1 = poor quality; 5 = excellent quality).

*General comments*
4. Please provide any other comments about the teleneurophysiology reporting session.

#### iii) Cost

Based on data gathered during the project the cost of extending the pilot to provide a full service with capacity for 700 EEGs per annum (including paediatric and adult patients) was estimated. This was compared with data from a specialty costing exercise, conducted by the finance department at Beaumont Hospital over the same period, to determine the unit cost per CN investigation with the traditional model of service delivery. Of note, the Beaumont Hospital unit cost was averaged over all CN investigation modalities including EEG, NCS, EMG and EP some of which may be more expensive than others. Non-pay as well as pay-related costs were examined and included equipment purchase, maintenance overheads, consumables, office supplies, organisational overheads, administration overheads, and salaries for consultant, technologist and administrative personnel.

### Data analysis

Quantitative data were tabulated and from this totals and proportions or percentages in different metric categories were established. Qualitative data from notes taken at the feedback group meetings, conversations and questionnaires were triangulated so that facts regarding technical performance and service provider satisfaction that emerged from each method were supported with findings from the other methods.

Finally, where possible, data from this study were contrasted with the previously reported analysis of the need for teleneurophysiology in the northwest of Ireland which included an audit of 4954 patient records from 6 Dublin-based CN departments over a one-year period as well as satisfaction questionnaires completed by 322 patients attending the same six centres [2,3]. These pre-intervention observations included data for both paediatric and adult patients, for all CN modalities (EEG, EMG, NCS, EP), and for patients resident in regions with and without a local CN service. As the pre and post-intervention patient groups were not equivalent, the pre-intervention data represent a control period rather than a control group [22] and provided some information for a preliminary comparison of the traditional and telemedicine models of CN service delivery. Descriptive statistics were used to summarise the comparison.

## Results

### Utilisation of teleneurophysiology service

Over a 20 week period, or 40 separate clinic sessions, 142 patients (74 female, 68 male) had an EEG investigation at SGH. Patients ranged in age from 18 to 86 years (patients below 18 years of age were excluded from the pilot study). 35% of the patients were between 18-30 years of age, 26% were between 31-50 years of age, 22% were between 51 - 70 years of age, and 17% were from 71 to 86 years of age. Ten percent (14/142) of the patients had a stated learning disability on their referral information. Based on their home address information, the average distance travelled by patients to SGH for the EEG investigation was 69 km (range from 1.6 km to 193 km). Thirty-two percent (45 patients) travelled a distance greater than 100 km.

Table 2. illustrates the specialty of clinicians who referred patients to the teleneurophysiology service. The top six referring specialties were medicine of old age (29/142), respiratory medicine (25/142), cardiology (21/142), psychiatry (19/142), internal medicine (17/142) and gastroenterology (14/142). It should be noted that the two neurologist referrals came from Beaumont Hospital based clinicians who had seen these northwest residents at Beaumont and for the patients' convenience had referred them to their local hospital in Sligo for EEG.

**Table 2:** Speciality of referring clinician and number of patients referred to the teleneurophysiology service.

Speciality	Number of Referrers	Number of Patients
**Medicine of Old Age**	4	29 (20%)
**Respiratory**	3	25(18%)
**Cardiology**	3	21 (15%)
**Psychiatry**	8	19(13%)
**Internal Medicine**	1	17 (12%)
**Gastroenterologist**	1	14 (10%)
**Physician**	2	7 (5%)
**General Practitioner**	1	5 (4%)
**Neurologist**	2	2 (1%)
**Paediatrician**	1	1 (1%)
**Surgery**	1	1 (1%)
**Nephrologist**	1	1 (1%)
**TOTAL**	**28**	**142 (100%)**

**Figure 2:**
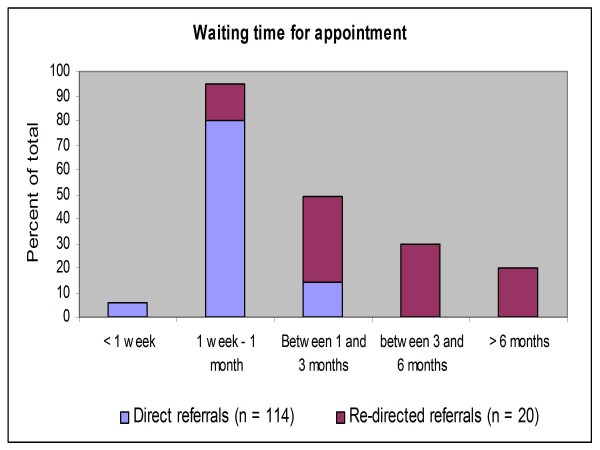
**Waiting time for teleneurophysiology appointment**. Some patients who were on waiting lists at other CN centres had their appointments re-directed to the teleneurophysiology centre.

The waiting time for appointment was calculated from the date of referral to the date of EEG appointment at SGH. Of the 142 patients seen at SGH, 122 were referred directly to the teleneurophysiology service. The remaining 20 were referrals re-directed from CN waiting lists in two other centres. These referrals had been made by northwest clinicians prior to the commencement of the teleneurophysiology project. Date of referral information was missing from 8 of the direct referral forms. Therefore, based on 114 patients the average waiting time for appointment was 22 days. Six percent had their EEG within one week of referral, 80% of appointments were between 1 week and 1 month from the date of referral and 14% waited longer than 1 month. However, referrals written and held in SGH in anticipation of the teleneurophysiology project accounted for half of those who waited longer than one month. An examination of the 20 re-directed referrals showed that by the time these patients had their EEG at SGH this group had an average waiting time of 140 days, with 30% of them waiting between 3 and 6 months and 20% waiting more than 6 months (See Figure 2).

The interpreting consultant categorised the EEG referrals into one of two groups; evaluation for either epileptiform abnormalities or mental state changes. Ninety-four percent (134) were assigned to the former, and 5% (7) belonged to the latter. One referral was thought to belong in both categories. Sixty-seven percent (95/141) of the EEG recordings were reported as normal, while 33% were found to have abnormalities. Forty-one percent (58) of the EEG reports were dictated, typed, verified and sent by post to the referring clinician within two weeks of the EEG appointment. A further 38% were sent within 1 to 2 weeks and the remainder were sent within 2 to 3 weeks.

### Technical performance and service provider satisfaction

Technical performance and service provider satisfaction was assessed through triangulation of data from questionnaires, one-to-one discussions and group meetings which were conducted over the course of the 20 week pilot study.

Although requested to complete feedback questionnaires at the end of each EEG recording session, in practice the technologists tended to be more purposeful in completing them. They completed questionnaires at the end of 12/40 (30%) EEG recording sessions corresponding to when there was something specific to report about a patient referral, an individual EEG, or a clinical session. For example, 6 completed questionnaires noted a lack of information on referral forms and 7 reported problems of electrical interference during EEG recording.

Feedback from the consultant neurophysiologist was largely provided through one-to-one discussion or at group meetings. He retrospectively completed those parts of the questionnaire concerning the nature and quality of referral details for 141/142 patients at the end of the pilot project.

#### a) Technical performance

Feedback from both technologists and the consultant neurophysiologist regarding the technical performance of the EEG recording and review equipment was very positive. Application software was found to be user-friendly. Some minor problems with electrical interference, photic stimulation and electrodes at SGH were easily solved with help from the equipment supplier. Re-arranging equipment in the EEG recording room significantly reduced electrical interference.

The CN local area network (LAN) at SGH was configured so that EEG recordings were automatically stored on the CN file server. A copy was also kept on the EEG acquisition computer as a back-up in the event of any network failure. No problems were reported regarding the reliability of the LAN.

The consultant neurophysiologist who interpreted the EEGs at Beaumont Hospital noted there were a lot of steps or mouse clicks required to access EEG files via the GVPN. These included logging onto the network using a user name and password and navigating to the shared data folder on the SGH file server. However, much of this process was required for security purposes to ensure that only authorised and authenticated personnel could access the patient data via the GVPN.

To review EEG data stored on the SGH file server, the consultant at Beaumont Hospital accessed the relevant file via the GVPN without transferring it between sites. The rationale for this approach was to avoid making unnecessary duplications of data and having to track different amended versions of patient files. Review of an individual patient's EEG required scrolling through approximately 200 ten second pages of EEG. A replay rate of about 2 pages per second is generally employed when reviewing an EEG. The rate can be varied up or down from this. Typically, during review the consultant will add annotations to the EEG file at points of clinical interest. These annotations become part of the record and are saved with the original EEG file. For patient safety, quality and data protection the authoritative version of the record (i.e original EEG recording and subsequent annotations) was stored centrally at SGH. Therefore, there was no permanent transfer of the EEG files between SGH and Beaumont.

It was noted that over the GVPN streaming of this data was occasionally slightly erratic. This was manifested by the replay working as expected for a number of pages and then freezing or an interruption to EEG data streaming. To alleviate the problem the reviewing consultant would close and re-open the file. While it caused some disturbance, this problem did not prevent EEG review and interpretation via the GVPN over the pilot project.

Over the 20 week EEG recording phase of the project, a total failure of the GVPN lasting 7 working days occurred. The problem was ultimately identified as being due to an external connection fault (i.e. in the telecommunications network outside either hospital). The delay in identifying the fault and re-establishing the connection was mainly one of communication between the different players involved in the provision of the GVPN. These included the IT departments at both hospitals, the telecommunication company that hosts the GVPN and two different firewall support companies engaged by the two hospitals.

The loss of GVPN connectivity required the implementation of a back-up arrangement. EEGs recorded just prior to and during the loss of connectivity were transferred on DVD to Beaumont Hospital for interpretation. Consequently no delay in the recording, interpretation and reporting process was experienced.

#### b) Service provider satisfaction

The consultant neurophysiologist examined 141 of the 142 referrals and found that 101 (72%) were made using the specified teleneurophysiology referral form. In 77 of these all sections of the form were complete. Forty referrals were made using a different referral form or by letter. Thirty-six (90%) of these were considered to have provided the required referral information. Twenty-eight (20%) referrals were deemed to have missing information, e.g. information about previous EEG studies, clear reason for referral, clinical summary and current medications.

Referrals were rated by the consultant on scale of 1 to 5 (where 1 = the referral was not appropriate for EEG investigation and 5 = the referral was highly appropriate for EEG investigation). Of the 139 referrals scored 40% (55) were graded 5, 55% (76) scored 4, and 6% (8) scored 3. None of the referrals were considered inappropriate.

The technologists reported that incomplete referral information had implications for the EEG investigation. For example, in the case of patients with a learning disability failure to provide adequate information could result in not enough time being allocated to facilitate EEG recording for a person who may have some difficulty co-operating with the investigation. Other than this, satisfaction of the technologists did not vary with patient characteristics.

The scheduling process was designed so that a daily timetable would be uploaded to the SGH file server by the scheduling assistant at Beaumont. In this way it would be readily available, preferably in advance of the EEG session, to the technologist and administrative assistant at SGH. On a small number of occasions, this process did not work as planned. In three instances when an appointment was cancelled a new patient was given the now vacant appointment slot. When this happened at short notice the modified schedule was not available in advance of the EEG session.

While individual EEG recordings were not graded, the consultant concluded that their overall quality was either very good or excellent (i.e. using a scale of 1 - 5 where 1 = poor, 2 = fair, 3 = good, 4 = very good, 5 = excellent). The majority of EEG recordings were considered to be grade 5 in quality.

Including the steps from turning on the EEG review computer, accessing a particular EEG file, opening and annotating the EEG, dictating a report, marking the file as reviewed and closing the file, the consultant indicated that on average 25 minutes were required to generate an EEG report.

Discussions regarding the telemedicine model of CN service delivery highlighted the need for on-going support and continued professional development for technologists working at a distance from their main department. For example, participants suggested that processes for ensuring quality of service must be considered and established. As the service crosses traditional organisational boundaries, systems for staff accountability and supervision that are appropriate to this structure need to be established. Mechanisms for communication between the technologist and reporting consultant need to be in place to facilitate discussion about particular EEG recordings and frequent joint reporting sessions. Periodic visits by the consultant to the satellite department and by the technologists to the host centre should also be made.

Feedback on the EEG recording environment was provided by the technologists. It was noted for example that the EEG recording room was not conducive to conducting sleep EEGs as the environment was sometimes not quiet enough. Occasionally conversations and other noise from adjacent clinic and waiting areas could be heard. Also, an intermittent problem of electrical interference reported on 7/12 of the technologist satisfaction questionnaires was considered to sometimes be environmental, possibly due to equipment being used in a neighbouring room.

### Cost analysis

A comparable unit cost per investigation was estimated using either the teleneurophysiology or traditional model of CN service delivery. During the same period that the teleneurophysiology pilot was conducted, a unit cost per investigation averaged over all CN modalities (EEG, EMG, NCS and EP) was found to be €531 with the traditional model of service delivery at Beaumont Hospital. Assuming a service capacity requirement of 700 EEGs per year for the northwest region of Ireland, a unit cost per EEG of €546 was projected using the teleneurophysiology model.

### Comparison of traditional and telemedicine CN models

Compared to an audit of traditional CN services in Ireland the teleneurophysiology utilisation data highlighted a number of issues [2]. There was a similar age distribution amongst the people who attended the teleneurophysiology service and northwest adult residents who previously had EEG investigations in CN centres in Dublin (age range: 18 - 86 years and 18 -78 years respectively). However, only 63 adults who were resident in the northwest region of Ireland were referred for EEG during the traditional service audit period. During the teleneurophysiology pilot 142 EEGs were recorded in 20 weeks representing more than a five-fold increase in access to EEG services for the population of that region. Similarly, average waiting times for investigation were halved from 6 to 3 weeks. A previous patient satisfaction survey recorded that people resident in a region of Ireland with no local CN service could travel a one-way distance of up to 320 km (average distances reported 146 km) for investigation [3]. By contrast the availability of the teleneurophysiology facility demonstrated the potential to significantly reduce this burden.

The earlier audit also showed that 41% of EEG referrals for northwest residents were made by neurologists whereas only 1% of the referrals to the teleneurophysiology service came from neurology with the remainder from a number of different medical specialties [3]. Conducted at a time when there were no specialist neurology services in the northwest, the audit indicated that people travelled outside their region of residence for medical consultation before being considered for EEG investigation. This implies additional hospital appointments, with additional waiting periods and long journeys. Taken together with the assessment that 95% of the referrals to the teleneurophysiology service were either highly or very appropriate, the suggestion is that some of these additional patient encounters with the health service may have been unnecessary.

## Discussion

This pilot project demonstrated the practicality of a telemedicine model for a CN service delivery. It further illustrated that a VPN for data exchange between Ireland's government agencies provided a secure and highly reliable link between Beaumont Hospital and SGH for electronic transfer of, and remote access to sensitive patient data. Participating CN technologists and consultants expressed their satisfaction with the teleneurophysiology model and made practical suggestions for enhancing it. In a previously reported analysis of need, distance and long waiting-lists seemed to deter clinicians in the northwest of Ireland from referring their patients for CN investigation [3]. Consequently, at that time the number of CN referrals from the region was 23% of expected [2]. During the teleneurophysiology pilot there was a five-fold increase in access to EEG investigation for the population of the northwest of Ireland together with a considerable improvement in waiting times. Furthermore, when compared to traditional CN the teleneurophysiology model of service delivery was found to be cost effective. While the study focussed on the delivery of an EEG service, there is potential to expand the model to include other neurophysiology modalities (e.g. EMG and NCS).

The current study did not aim to demonstrate causality between the teleneurophysiology model of service delivery and service outcome [22]. Therefore, in comparing the pre and post-intervention data descriptive rather than inferential statistics were employed. This study was a formative assessment to examine how the demands of the social and technical dimensions of the model fit with each other and with the organisational environment [26]. In this regard, observations on utilisation, technical performance and CN personnel satisfaction can inform requirements for further development and governance of teleneurophysiology services. The following discussion of our results therefore considers those aspects of the model that worked well and those that present opportunity for improvement.

The technical dimension (including the EEG recording and review equipment, the CN file server and LAN, and the GVPN) of the teleneurophysiology model performed well. However, a loss of GVPN over 7 working days together with unexplained sporadic glitches in replaying EEG over the GVPN highlighted the need for reliable IT support at both the host and satellite sites. Responsibility for maintaining continuous connectivity to local and wide area networks (e.g. GVPN) should be unambiguous to prevent delays in dealing with problems. For example, it was found that a number of different entities can be involved in the provision of the GVPN - a GVPN host company, firewall support companies and local IT departments. In the event of network problems, responsibility for troubleshooting and repair must be unequivocal.

A back-up arrangement of copying EEG files to DVD and then transferring the DVD to Beaumont Hospital was put into action to deal with the temporary failure of the GVPN. Patient confidentiality was maintained as specialised software was required to open and review EEG files. Nevertheless, as previously outlined, the preferred solution is to store an authoritative single version of the file centrally from where it can be accessed for review via a secure VPN by authorised and authenticated clinicians.

A combination of objective facts (in completed questionnaires) and subjective accounts (at group feedback meetings and one-to-one conversations) were collected from the service providers. Although questionnaires were not completed at the end of each EEG recording session or review session as intended, the triangulation of these methods captured a richness of information to illustrate the strengths and weaknesses of the teleneurophysiology mode of work.

The consultant neurophysiologist did not consider any of the EEG requests to the teleneurophysiology service to be inappropriate and the ratio of abnormal to normal EEG recordings (1:2) found over the twenty week pilot period is typical for routine adult EEG services with similar referrer profile [27]. Even where there is a known seizure disorder a routine EEG recording, which records a 20-40 minute sample of the electrical activity of the brain, may not demonstrate an abnormality. As evidenced by the quality of referrals, the performance of the scheduling process, the quality of EEG recordings, and the relative ease of EEG review and reporting, no significant problems arose in the teleneurophysiology protocols and procedures. However, some observations suggest that the physical separation of different parts of the teleneurophysiology service may amplify the challenges of the social dimension of service delivery. In either setting, telemedicine or traditional, incomplete referral information can be a problem for the service provider. It has the potential to result in an inappropriate approach to EEG recording if indication for EEG is not clear or readily available, delays for the patient and other time management difficulties. For example, patient scheduling took place at the host site which was 130 miles form the satellite centre. Our evaluation showed that information was missing from 20% of referrals and incomplete schedule information was conveyed to the satellite on a small number of occasions. When technologists work remotely, overcoming these issues may be more challenging as the same support will not be available in the satellite setting as are in the host centre. Assistance for tracking more complete referral information may not be available. Similarly, mechanisms for interaction between scheduling and technical personnel may be such that suboptimal schedules of work are arranged. The implication is that higher standards in aspects of operations management compared to the traditional single site model may be required (e.g. zero-tolerance for incomplete referral information).

The participants in this study recognised a need to establish clear mechanisms of communication between the technologists in the remote centre and the consultant neurophysiologist in the host centre. These mechanisms for communication need also to consider communications between all clinical and non-clinical (e.g. scheduling assistant) personnel who are involved in the delivery of the service. In this regard, the model could be enhanced by the addition of video-conferencing infrastructure so that geographically separated personnel could participate together in departmental meetings, scheduling sessions, case conferences, training, lectures, and joint reporting sessions [28,29].

Temporary EEG recording accommodation at SGH was used in this pilot study. The feedback on this environment indicates a need to adopt appropriate standards when commissioning CN rooms including the necessity for a quiet location to facilitate EEG testing, freedom from electrical interference from adjacent departments, close proximity to other clinical staff for support in the event of an emergency, and access to emergency equipment.

Commentators on telemedicine, covering a wide range of technologies and application, say that its effectiveness has yet to be researched and that in general telemedicine literature contains examinations of structure and process with very little on outcome [12-20]. Currell et al., (2000) warn that without systematic evaluation healthcare policy makers and managers should be cautious about recommending increased investment in unevaluated technologies [30]. The teleneurophysiology evaluation presented here reports on structure and process, and explores the short-term outcome of the model. A longer time-frame of evaluation would be required to demonstrate any causal link between the intervention and effects on patient care and management. For example, a randomised controlled trial (RCT) to reliably assess effect and efficiency should only be conducted when the system has achieved reasonable stability [21,31,32].

## Limitations

The preliminary nature of this pilot project, its limited duration and bounded conditions, and the bias inherent in some of the assessment must be acknowledged. Although previous audit and satisfaction studies provided some pre-test data [2,3], it cannot be claimed that the improved EEG referral rate and waiting times compared to conventional Irish CN service are attributable to the teleneurophysiology model. Rather these changes may be due to pent-up demand for care in an under-served region [16]. Neither can it be assumed that the long-term outcomes of the model would remain positive.

It cannot be claimed that the small sample of participants (2 neurophysiological measurement technologists and 1 consultant in CN), who were purposefully selected to participate, and delivering the service over a twenty week period is representative of a larger population and longer duration. Therefore our results may not be generalisable.

Given the available technology and network, a limitation in the teleneurophysiology process was that hardcopy referral forms and regular mail for conveying reports were used. These requirements were due to wider organisational processes that were outside the control of this project.

The cost analysis presented is preliminary and has a number of constraints. For example the unit cost of a tele-EEG was compared to a unit cost of traditional CN investigation averaged over all CN modalities including EEG, EMG, NCS and EP. Furthermore, indirect costs, such as travel and loss of time at work or in their community for the patient, were not considered. A complete economic analysis would require control of confounding variables and inclusion of all costs.

## Conclusion

In conclusion, the potential of telemedicine to provide a more equitable CN service by supporting the decentralisation of access to specialist care has been demonstrated [33]. Implementing the system requires the EEG equipment suppliers to liaise with IT specialists to establish the required network interfaces. Once these are in place on-going maintenance and quality assurance of the EEG recording and review equipment will be conducted according to the normal practice in place in the traditional clinical neurophysiology department. In addition to this IT support must be available to ensure reliability of the VPN between the host and remote site. Identified opportunities for improving the teleneurophysiology model illustrate that when a service crosses traditional organisational boundaries, its regulation and management must reflect this [34]. Clarity in terms of individual and organisational roles and responsibilities is required (e.g. clinical governance, custody of clinical records, data protection and security, continued professional development, safety and risk management, human resource management, EEG equipment management, IT and network support, budget management and cost apportionment) so that appropriate attention is given to both the social and technical aspects of the model.

## Competing interests

The authors declare that they have no competing interests.

## Authors' contributions

PB, KM, GB, FM and MF were involved in the conception and design of the study, the collection of data, its analysis and interpretation. The process of the study and interpretation of data were further reviewed and monitored in detailed discussion with VR, CD, ND and SC. The manuscript was prepared for publication by PB and MF with critical review from the remaining authors. All authors approved the final version.

## Pre-publication history

The pre-publication history for this paper can be accessed here:

http://www.biomedcentral.com/1472-6947/10/48/prepub
